# Identifying Best Practices for Recruiting Adults with Limited Formal Education to ADRD Clinical Trials: Findings from a Systematic Scoping Review

**DOI:** 10.1002/alz70860_106297

**Published:** 2025-12-23

**Authors:** Allison Gibson, Dimitra Tziarli

**Affiliations:** ^1^ St. Louis University, St. Louis, MO, USA; ^2^ Saint Louis University, St. Louis, MO, USA

## Abstract

**Background:**

In order to ensure ADRD clinical trials reflect the real‐world population, it is essential to involve those with limited formal education (“completed secondary school” or less). Currently, most ADRD clinical trials are over‐representative of persons with college degrees and those from higher socioeconomic backgrounds. Those with limited formal education often experience higher rates of chronic diseases and poorer health outcomes. As such, they often have may have different health risks, treatment responses, and socioeconomic challenges that impact health outcomes. Further, ethical guidelines emphasize inclusivity in research to avoid bias and ensure equitable distribution of potential benefits and risks of new treatments. The aim of this study was to explore extant research on best practices for recruiting adults to ADRD clinical trials with limited formal education.

**Method:**

A systematic scoping review of scholarly literature was conducted using the search terms “low education level” OR “limited formal education” OR “low literacy” OR “lower educational attainment” AND “clinical research participation” OR “enrollment in clinical trials” to locate articles in Academic Search Complete, ScienceDirect, Wiley Online, Cochrane Database of Systematic Reviews, PubMed databases. Scholarly articles in peer reviewed journals were deemed eligible if they provided recommendations for addressing barriers or supporting facilitators to clinical research participation.

**Result:**

Twenty‐eight articles met these criteria and were included in the systematic scoping review. Findings suggest that, despite limited knowledge on this topic, there is evidence that participants with limited formal education require targeted communication and relationship building. Further, many of these participants may experience additional barriers to participate in trials.

**Conclusion:**

Recommendations for targeted communication will be presented. Trials were particularly effective when they built trust in the community through the process of educating about the research process and the importance of clinical research for their community. Successful studies also recognized community‐specific barriers to study participation and utilized innovative approaches to overcome them.

